# Maladaptive task-unrelated thoughts: Self-control failure or avoidant behavior? Preliminary evidence from an experience sampling study

**DOI:** 10.3389/fpsyt.2023.1037443

**Published:** 2023-03-14

**Authors:** Monika Kornacka, Michał S. Skorupski, Izabela Krejtz

**Affiliations:** ^1^Emotion Cognition Lab, SWPS University of Social Sciences and Humanities, Katowice, Poland; ^2^Insitute of Psychology, SWPS University of Social Sciences and Humanities, Warsaw, Poland

**Keywords:** task-unrelated thoughts, avoidance, self-control, emotion regulation, rumination

## Abstract

**Introduction:**

Task-unrelated thoughts (TUT) play an important role in everyday life functioning (e.g., anticipating the future, or providing a mental break). However, TUT might also be maladaptive, impairing cognitive performance emotion regulation, and increasing the risk of psychological disorders. In the present study, we aimed to test how self-reported control over TUT and task valence moderate the link between task difficulty and TUT intensity, testing the context regulation and avoidant alternative hypotheses of TUT occurrence.

**Method:**

Forty-nine participants took part in an experience sampling study. They were asked to answer five times a day for 5 days a series of questions assessing the intensity, valence, control over TUT, and their momentary affect along with characteristics of the task they were currently performing. They also filled in trait questionnaires assessing their tendency to daydream, ruminate, and their beliefs on emotions' usefulness and controllability.

**Results:**

The results showed that both task difficulty and one's lower control over thoughts along with their interaction significantly increased TUT intensity. Task negative valence significantly predicted TUT intensity and moderated the link between task difficulty and TUT intensity. In addition, the tendency to daydream and beliefs in the controllability of negative emotions affect the relations in this model.

**Discussion:**

To the best of our knowledge, this study is the first to provide quantitative evidence from an experience sampling study on the role of the valence of currently performed tasks and beliefs on emotions on TUT intensity. It might be an important indication for research and clinical practice that maladaptive TUT might not be only linked to self-control failure but also to emotion regulation strategies one is using.

## Introduction

Task-unrelated thoughts (TUT), defined as an engagement in mentation that occurs unintentionally and is unrelated to one's current activity and surroundings (1), are considered a default mental activity occurring on a daily basis (2). Some studies suggest that off-task thinking might take more than one-third of our waking activity (3, 4). TUT play numerous adaptive functions, from planning and anticipating the future and enhancing goal progress to providing a break from difficult or boring activities to increasing creativity by letting our mind move freely to new directions (5–8). However, other studies suggest that task-unrelated thoughts, under certain circumstances, might also be maladaptive and not only lower current task performance [for a review, refer to: (7)] but also impair emotion regulation and increase the risk of psychological disorders (9–11).

The literature suggests several hypotheses to explain these potential maladaptive outcomes of TUT, for example, the context regulation theory (2) and the avoidant alternative hypothesis (12). The context regulation theory (2) suggests that adaptive TUT occur when an individual performs a task not requiring a full engagement of cognitive resources. However, when the task becomes cognitively demanding, one should recruit executive resources to stop off-task thinking and focus on the ongoing activity. Thus, the adaptive feature of TUT would depend on the interaction between contextual factors (task difficulty) and personal disposition (executive resources), thus, TUT occurrence during a demanding task can manifest executive resources failure.

However, the literature suggests that the TUT occurrence during difficult tasks might depend not only on self-control resources but also on TUT function (12). TUT can be also an avoidant alternative for a difficult or distressing task. In this case, TUT occurrence during a difficult and cognitively demanding task might not (or not only) depend on the self-control resources but also on the emotion regulation strategy one is using (i.e., escaping from a distressing task into daydreaming). Thus, we can hypothesize that TUT intensity would be higher when one is performing a difficult, negatively valence task compared to the task of positive or neutral valence.

Although the involvement of executive resources in TUT is relatively well described in empirical studies, particularly those conducted in laboratory settings [e.g., (2, 13, 14)], the TUT as an avoidance mechanism remains described only in theoretical models and few qualitative studies [e.g., (12, 15)]. The present experience sampling study's aim was 2-fold. First, we tested the role of subjective control over one's TUT in the link between task difficulty and TUT level during participants' everyday activities testing the context regulation theory (2). Second, we examined the role of task valence in the link between task difficulty and TUT level testing the avoidant alternative hypothesis (12).

### Role of executive resources and thoughts control in TUT

Several laboratory and experience sampling studies showed that executive functions might be involved in the maintenance of unintentional task-unrelated thoughts often impairing one's performance in the current task [e.g., (1, 16–18)]. In line with the context regulation theory, Kane and McVay (2) suggested that cognitive abilities might interact with the situational context (e.g., task characteristics) to determine the adaptive feature of TUT. Rummel and Boywitt (14) brought up evidence through a laboratory study that working memory capacity enables participants to adjust their TUT level to situational demands. Moreover, the link between executive functions and task-unrelated thoughts might also depend on the ongoing task difficulty. The relation between executive resources and TUT intensity is positive when the task is not demanding, and negative when it requires cognitive resources—in this case, executive functions help to adjust TUT intensity to situational demands. In a recent study, Marcusson-Clavertz et al. (19) showed that executive functions measured in the laboratory predict the TUT intensity in experience sampling measures—better updating leads to a decrease in TUT when one is trying to focus on the task in everyday life.

Surprisingly, Barrington et al. (20) remark that some studies showed that mind-wandering (MW) can also increase with task difficulty. One possible explanation might be that the relationship between task difficulty and TUT level can take the form of a U-shape, and the TUT level is relatively high when the ongoing task is easy and decreases with the task difficulty but only to a certain point when the task becomes too difficult and TUT starts to increase again due to the cognitive overload (21). However, Barrington et al. (20) found, in a set of two laboratory studies, that the relationship between task difficulty and TUT intensity might not depend only on objective executive resources efficiency but also on subjective evaluation of the task (e.g., a subjective difficulty or motivational factors).

### Avoidance role of task-unrelated thoughts

The involvement of motivational factors in the level of TUT during difficult tasks seems to be congruent with another line of research on TUT maladaptive outcomes, suggesting that TUT might be an emotion regulation strategy based on avoidance. In a qualitative study, Somer (12) showed that one of the main functions of TUT might be avoiding emotionally difficult or distressing experiences. When the task is distressing, TUT might be used as a form of experiential avoidance, and thus, its level might increase even if the ongoing task is demanding (12). The hypothesis of TUT as an avoidant alternative to a distressing task seems to be endorsed by both theory and empirical evidence linking TUT to affect. First, the theoretical support of the TUT avoidance function comes from the theory of repetitive negative thinking. Although there is still no consensus whether adaptive mind-wandering or daydreaming and rumination might be two opposite end points on the continuum of task-unrelated thoughts [see: (22, 23)], repetitive negative thinking is often considered a “sticky form” of off-task thinking (24). This similarity is particularly visible in light of the goal theory of current concerns (25), which suggest that in a situation when goal progress is not available through operant behavior, the goal striving will occur as a purely cognitive response (i.e., mind-wandering or daydreaming about that goal/concern). A diary study of daydreaming by van Rijn et al. (26) showed that daydreaming seems to incorporate one's current concerns from the 2 previous days. In line, Martin and Tesser (27) in their control theory of rumination suggest that this kind of cognition will be triggered by actual-ideal self-discrepancy resulting from an unresolved personally relevant goal. Second, in the field of repetitive negative thinking theory, Thomas Borkovec developed the avoidance theory of worry (28) suggesting that paradoxically worrying might serve as a cognitive avoidance response to perceived threats. In one of the rare experimental studies, Giorgio et al. (29) tested experimentally the hypothesis of rumination as avoidant behavior but without conclusive results. They found that trait rumination is linked to self-reported avoidant behavior, but those results were not replicated in the laboratory avoidance task (29).

Moreover, previous studies suggested that negative affect might trigger TUT [e.g., (30)]. Kane et al. (31) showed also that anxious individuals characterized by avoidant behavior tend to have a higher level of mind-wandering. Apart from a qualitative study by Somer (12) showing that MW is associated with anxious avoidance and that this behavior might be maintained by negative reinforcement—a mechanism classical for anxiety disorders—a recent experience sampling study (32) showed that participants under chronic stress reported more MW. In addition, they found that those participants also tended to reject their current experience more. Those results seem to be particularly interesting in the context of TUT as experiential avoidance, suggesting also that the willingness to accept negative emotion or one's beliefs on how helpful or controllable negative emotions are (33) might play a role in a potential avoidance function of TUT.

However, in spite of a relatively strong theoretical background, the avoidance role of task-unrelated thoughts is very rarely experimentally tested—one of the main reasons might be the lack of a direct and valid measure of avoidance. On the one hand, self-reported measures of avoidance are available [e.g., (34)]; nevertheless, it seems that most people struggle to identify this function of their cognition; thus, self-reported measures are subject to biases. On the other hand, many studies measure avoidance in laboratory conditions [e.g., using an approach–avoidance task; for a review, refer to (35)]; however, these kinds of measures are often not ecologically valid and seem to be not applicable to measure TUT function. Thus, in the context of TUT appearing in participants' daily life, the first step to explore the potential role of avoidance in TUT occurrence seems to be testing whether task difficulty, but also task valence, might be linked to TUT occurrence. If TUT plays an avoidance function, it should occur not only during an easy task, but its level should be higher also when the task has a negative valence, compared to the task with positive valence.

### Aim of the present study

In the present study, we aimed to test how self-reported control over TUT and task valence moderate the link between task difficulty and TUT intensity in participants' everyday lives, testing both the context regulation and avoidant alternative hypothesis of TUT occurrence. In addition, we checked whether trait measures—general tendency to daydream, ruminate, and beliefs about negative emotion—can affect those relations. An important strength of the present study was to test all the variables using the ecological momentary assessment (EMA). The use of ESM is especially relevant when studying changes in affect and thought content, as both of these phenomena change dynamically throughout the day and can be more prone to misrepresentation when probed with retrospective methods than with repeated measurements in an ecological environment (36, 37).

## Methods and materials

### Participants

Seventy-two volunteers from a community sample took part in the study on day 1 by filling in the online trait questionnaires. Sixty-two participants agreed, at the end of the questionnaire part, to follow up with the experience sampling part of the study and installed the MovisensXS application (38) on their mobile phones. The participants with compliance rates lower than 30% in the EMA part of the study were excluded from the analyses. The use of this criterion is popular in EMA studies (39), as participants' compliance under 30% is considered poor (40, 41) and potentially unreliable (42, 43). This resulted in the final sample consisting of 49 participants (mean age = 30.73, SD = 5.82, 38.8% women). Those 49 participants provided 862 momentary assessments with a mean compliance rate of 70.04%, which can be considered an acceptable compliance rate for short, 4–6 days, experience-sampling studies according to recent recommendations (44).

### Materials

#### Trait measures

##### Mind-wandering

Trait MW was evaluated through Daydreaming Frequency Scale [DDFS; (45–47)]. This 12-item self-reported questionnaire assesses the general frequency of stimulus-independent and task-unrelated thoughts. We used the Polish version of DDFS (48), which has excellent internal consistency (Cronbach's α = 0.92) and good criterion validity. Cronbach's α in the present study was 0.93.

##### Repetitive negative thinking

Trait RNT was evaluated through the transdiagnostic Perseverative Thinking Questionnaire [PTQ; (49)]. This 15-item questionnaire assesses the main features of RNT (unproductiveness, repetitive features, and mental capacity captured by RNT) from a transdiagnostic disorder-independent perspective. The internal validity of the RNT score was satisfying with Cronbach's α of 0.92.

##### Beliefs on emotion

Beliefs on emotion were assessed through the Emotion Beliefs Questionnaire (50). The questionnaire assesses general beliefs on emotions along with the usefulness and controllability subdimension for both positive and negative emotions. It is worth noting that higher scores on the usefulness subscale mean stronger beliefs about the uselessness of certain emotions, and higher scores on the controllability subscale mean stronger beliefs about the uncontrollability of emotions. The internal validity of the questionnaire was satisfying with Cronbach's α of 0.87.

#### EMA measures

##### TUT intensity and characteristics—control and valence

TUT evaluation was adapted from Kornacka et al. (51). Participants were asked three questions assessing the task-unrelated character of their thoughts: “Just before the bip, (1) to what extent you were focused on your current main task” (not at all–totally); (2) “you had control over your thoughts” (not at all–totally); and (3) “what was the valence of your thoughts” (negative–positive). They provided answers on a visual analog scale (VAS) from 0 to 100.

##### Context–task characteristics

Task characteristic evaluation was adapted from Granholm et al. (52). Participants were asked to characterize their ongoing task by answering the following questions: “To what extent the task you are currently performing is”: (1) difficult; (2) interesting; and (3) pleasant. They provided answers on the VAS from 0—not at all, to 100—totally. Those questions evaluate the main task characteristics that might be crucial in the context of TUT according to the theoretical context of the present study. Evaluating task characteristics instead of the ongoing task type (e.g., leisure, work, family time, as in some of the previous studies) seems to be important, particularly in the context of exploring TUT using EMA—in the ecological condition, the same/similar task but performed in the other context or by another individual might have different subjective valence and difficulty. Thus, the task type seems to be less informative, and evaluating task characteristics directly seems to be more relevant.

##### Mood

Mood evaluation was adapted from Pe et al. (53). Participants were asked to evaluate on the VAS scale from 0 (not at all) to 100 (totally) to what extent they felt happy, interested, anxious, sad, or angry.

### Procedure

Participants were recruited online through social media. They were informed that they would take part in a daily sampling study on daydreaming. They expressed informed consent and filled in online trait questionnaires on day 1.^1^ They were contacted by an experimenter, and the daily sampling procedure was explained. All the EMA data were collected through the MovisensXS application installed on participants' personal mobile phones. On days 2–6, participants responded to five signals a day randomly sent in daily activity slots (from 8 a.m. to 10 p.m.). The minimal interval between the signals was set up to 60 min. The study was run fully remotely, without any financial gratification for the participants, and was approved by a local ethics committee (WKEB69/03/2021).

### Statistical analysis plan

The interval-contingent data collected in the present study should be analyzed with multilevel random coefficient modeling. Each momentary entry will be nested within each individual. In order to examine whether subjective control over one's thoughts and task valence moderate the link between task difficulty and TUT intensity, and to further explore the moderating role of trait daydreaming tendency and metacognitive beliefs on emotion, we used multilevel models analyses, computed in R [version 4.2.1; (54)] with the “lme4” package [version 1.1-30; (55)].

Task characteristics (difficulty, valence) and TUT characteristics (subjective level of control over them) were treated as level 1 predictors and nested in participants (level 2). Trait-level variables measured with Daydreaming Frequency Scale, Emotional Beliefs Questionnaire, and PTQ were entered separately into the model described earlier as level 2 predictors. Before entering the analyses, all level 1 variables were group mean-centered and level 2 variables were grand mean-centered. The descriptive statistics and correlations for the level 1 and 2 variables are presented in Table 1.

**Table 1:** Descriptive statistics and correlations of level 1 and 2 variables.

**Level 1 variables (*****N** =* **49)**								
	**Descriptive statistics**		**Correlations**					
**Variable**	**Mean**	**SD**	**1**.	**2**.	**3**.	**4**.	**5**.	
1. TUT intensity	32.77	32.85	-					
2. Thought control	69.42	28.09	−0.66^***^	-				
3. TUT valence	71.75	26.27	−0.33^***^	0.42^***^	-			
4. Task difficulty	26.15	29.32	0.25^***^	0.20^***^	−0.14^***^	-		
5. Task interest	59.38	32.27	−0.41^***^	0.34^***^	0.37^***^	0.23^***^	–	
6. Task valence	67.87	26.78	−0.23^***^	0.24^***^	0.53^***^	−0.24^***^	0.57^***^	
**Level 2 variables (*****N** =* **49)**								
	**Mean**	**SD**	**7**.	**8**.	**9**.	**10**.	**11**.	**12**.
7. DDFS	38.51	9.26	-					
8. PTQ	44.39	10.21	0.32^*^	-				
9. EBQ total score	31.32	11.97	0.10	−0.05	-			
10. EBQ negative controllability	9.02	3.96	0.17	0.12	0.81^***^	-		
11. EBQ positive controllability	9.77	5.09	0.09	−0.06	0.80^***^	0.70^***^	-	
12. EBQ negative usefulness	7.81	4.72	−0.05	−0.13	0.71^***^	0.23	0.30^*^	-
13. EBQ positive usefulness	4.87	2.30	0.06	−0.03	0.59^***^	0.38^**^	0.12	0.57^***^

DDFS—Daydreaming Frequency Scale, PTQ—Perseverative Thinking Questionnaire, EBQ—Emotional Beliefs Questionnaire. Correlations for level 1 variables were computed using the repeated measures correlation method.

* *p* < 0.05;

** *p* < 0.01;

*** *p* < 0.001.

A likelihood-ratio test was used to compare all the multilevel models tested. A deviance drop comparison to an unconditional model was also computed. The unconditional model (with no predictors) for TUT intensity as an outcome variable is presented as follows:


$$\begin{array}{l} {TUT\;intensity}_{\text{ij}}=\gamma_{00}+u_{0\text{j}}+\;r_{\text{ij}}. \end{array}$$

The multicollinearity of variables included in all models presented in the following section was tested by computing variance inflation factors (VIFs). As the VIF values never exceeded 2.5, no multicollinearity has been detected (56, 57).

## Results

### Testing the context regulation hypothesis

First, we tested how task difficulty and thought control and their interaction predict TUT intensity using the model specified as follows:


$$\begin{array}{l} TUT\;intensity_{\text{ij}}=\gamma_{00}+u_{\text{0j}}\;+\;\beta_{1j}(Task\;difficulty_{ij}{)}_{+}\beta_{2j}(Thought\;\\ \;control_{ij})+\beta_{3j}(Thought\;control_{ij}\;\times \;Task\;difficulty_{ij})+\;r_{\text{ij}} \end{array}$$

$$\begin{array}{l} {\text{β}}_{1\text{j}}=\gamma_{10}+u_{1\text{j}}\\ {\text{β}}_{2\text{j}}=\gamma_{20}+u_{2\text{j}}\\ {\text{β}}_{3\text{j}}=\gamma_{30}+u_{3\text{j}}. \end{array}$$

The results suggest that both task difficulty and thought control are statistically significant predictors of TUT intensity. As we anticipated on the basis of the context regulation hypothesis, a rise in both task difficulty and thought control was related to the decline of TUT intensity (see Model 1 in Table 2).

**Table 2:** Testing level 1 predictors and their interactions link to momentary TUT intensity.

	***Coeff***	***SE***	***t*-value**
**Model 1—Task difficulty and thought control interaction**			
Task difficulty	−0.15	0.03	5.03^***^
Thought control	−0.79	0.03	23.47^***^
Task difficulty x thought control	0.003	0.001	2.02^*^
Deviance drop compared to unconditional model			488.1
Significance of likelihood ratio test			*p* < 0.001
**Model 2—Task difficulty and task valence interaction**			
Task difficulty	−0.36	0.04	9.85^***^
Task valence	−0.39	0.04	9.59^***^
Task difficulty x task valence	0.004	0.001	2.97^**^
Deviance drop compared to unconditional model			146.2
Significance of likelihood ratio test			*p* < 0.001

* *p* < 0.05;

** *p* < 0.01;

*** *p* < 0.001.

**Figure 1:**
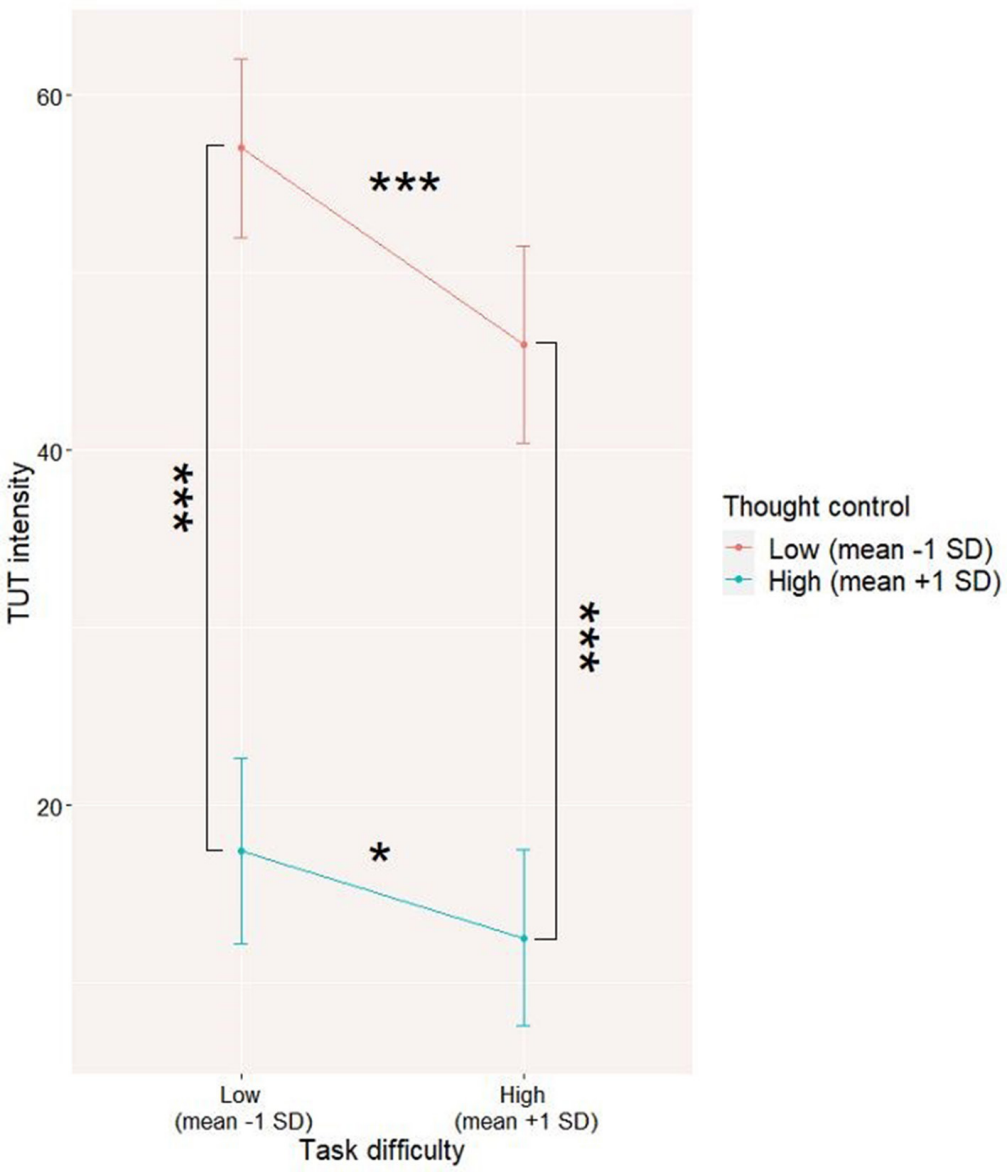
Level 1 interaction between thought control and task difficulty on task-unrelated thoughts (TUT) level. The interaction was visualized by computing slopes for high (mean +1SD) and low (mean-1SD) levels of predictor and moderator. *p < 0.05; ***p < 0.001.

The interaction turned out to be also significant (refer to Model 1 in Table 2), with a steeper slope (*Coeff* = −0.22, *t* = 4.88, *p* < 0.001) for low thought control than for high thought control (*Coeff* = −0.08, *t* = 2.17, *p* < 0.05), suggesting that the link between TUT intensity and task difficulty is stronger in situations where a participant has less control over thoughts (refer to Figure 1). Although this result might seem surprising, the data suggest that participants with lower control over their thoughts have significantly higher levels of TUT compared to participants with better control over their thoughts. Difficult task causes a drop in the TUT level, and this drop is larger for participants with a lower level of control over their TUT; however, their level of TUT still remains significantly higher compared to participants with good control over their thoughts (refer to Figure 1).

### Testing the avoidant alternative hypothesis of TUT occurrence

To test how task difficulty and task valence along with their interaction predict momentary TUT intensity, we used the model specified as follows:


$$\begin{array}{l} TUTintensity_{\text{ij}}=\gamma_{00}+u_{\text{0j}}+\beta_{1j}(Task\;difficulty_{ij}{}_{)}+\\ \beta_{2j}(Task\;valence_{ij})+\beta_{3j}(Task\;difficulty_{ij}\;\times \;Task\;valence_{ij})+\;r_{\text{ij}} \end{array}$$

$$\begin{array}{l} {\text{β}}_{1\text{j}}=\gamma_{10}+u_{1\text{j}}\\ {\text{β}}_{2\text{j}}=\gamma_{20}+u_{2\text{j}}\\ {\text{β}}_{3\text{j}}=\gamma_{30}+u_{3\text{j}}. \end{array}$$

The results (Model 2 in Table 2) suggest that both task difficulty and task valence are statistically significant predictors of TUT intensity. A rise in task difficulty and a more positive valence of the task were both related to a decline in TUT intensity.

**Figure 2:**
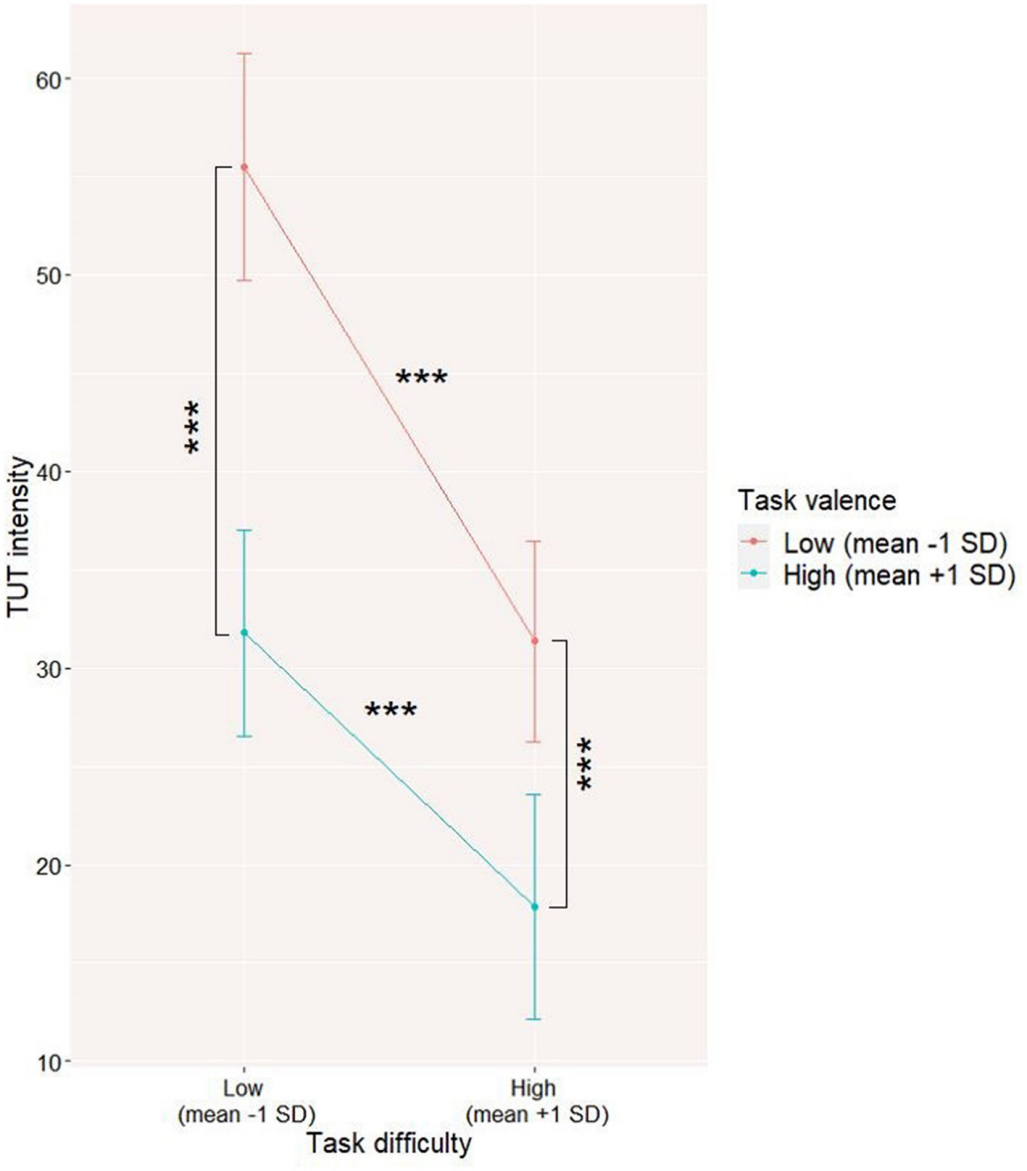
Level 1 interaction between task valence and task difficulty on task-unrelated thoughts (TUT) level. The interaction was visualized by computing slopes for high (mean +1SD) and low (mean-1SD) levels of predictor and moderator. ***p < 0.001.

We tested the same predictors with an added interaction term (task difficulty x task valence). The interaction turned out to be significant (refer to Model 2 in Table 2), with a steeper slope (*Coeff* = –0.48, *t* = 9.60, *p* < 0.001) for negative valence than for positive task valence (*Coeff* = −0.28, *t* = 5.28, *p* < 0.001), suggesting that the link between TUT intensity and task difficulty is stronger for less positive tasks; however, the TUT level remains significantly higher when the task is unpleasant for both easy and difficult tasks (refer to Figure 2).

### Trait characteristics as moderators of level 1 interactions

We then tested how trait characteristics moderate the aforementioned models. To do this, we incorporated the trait measures separately into Models 1 and 2 as level 2 predictors. In each of the models presented in the following equation, a single level 2 variable was added to the model, as in Model 1a presented in the following equation:


$$\begin{array}{r} TUTintensity_{\text{ij}}=\gamma_{00}+u_{\text{0j}}+\beta_{1j}(Task\;difficulty_{ij}{}_{)}+\\ \beta_{2j}(Task\;valence_{ij})+\beta_{3j}(Task\;difficulty_{ij}\\ \times \;Task\;valence_{ij}\;\times \;DDFS_{ij})+r_{\text{ij}} \end{array}$$

$$\begin{array}{l} {\text{β}}_{1\text{j}}=\gamma_{10}+u_{1\text{j}}\\ {\text{β}}_{2\text{j}}=\gamma_{20}+u_{2\text{j}}\\ {\text{β}}_{3\text{j}}=\gamma_{30}+u_{3\text{j}}. \end{array}$$

**Figure 3:**
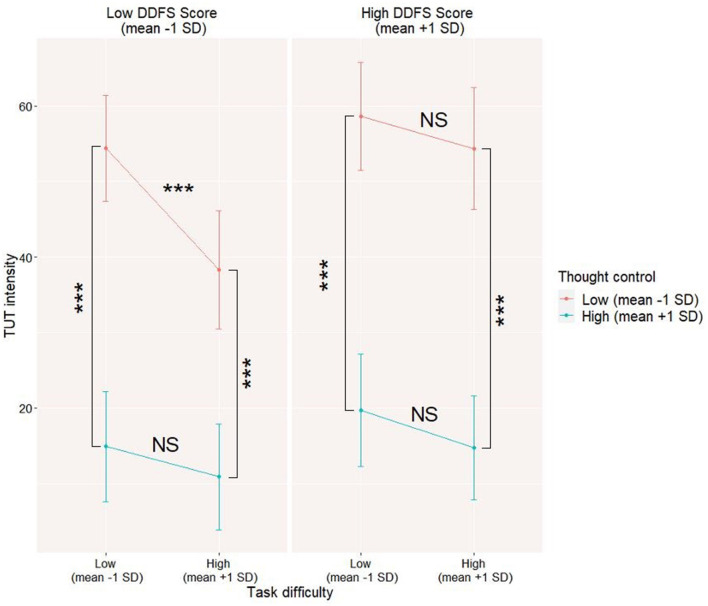
Daydreaming Frequency Scale (DDFS) moderating level 1 interaction between thought control and task difficulty on task-unrelated thoughts (TUT) level. The data for the moderation model were visualized by computing slopes for high (mean +1SD) and low (mean-1SD) levels of each predictor and moderator. NS—not significant; ***p < 0.001.

First, we constructed the models testing the moderation of the task difficulty x thought control interaction by trait-level characteristics. Only the DDFS score seems to moderate the level 1 interaction between thought control and task difficulty (refer to Model 1a in Table 3). It seems that TUT level decreases during difficult tasks compared to the easier ones but only for participants with a low level of thought control and a low level of trait tendency to daydream. This effect predicted by the context control hypothesis is not observed in frequent daydreamers (refer to Figure 3). Neither EBQ total score nor EBQ subscales or PTQ score turned out to be significant moderators of the interaction slope (refer to Models 1b−1e in Table 3).

**Table 3:** Testing level 2 variables as moderators of level 1 interactions in Model 1.

**Models based on Model 1**			
	***Coeff***	***SE***	***t-*** **ratio**
**Model 1a—DDFS score as level 2 moderator**			
Task difficulty	0.01	0.003	1.72
Thought control	−0.01	0.004	1.98^*^
Task difficulty x thought control	−0.0003	0.0001	2.08^*^
Deviance drop compared to unconditional model			497.93
Significance of likelihood ratio test			*p* < 0.001
**Model 1b—EBQ score as level 2 moderator**			
Task difficuly	−0.003	0.003	1.01
Thought control	0.005	0.003	1.47
Task difficulty x thought control	0.0001	0.0001	0.94
Deviance drop compared to unconditional model			493.22
Significance of likelihood ratio test			*p* < 0.001
**Model 1c—EBQ negative controllability score as level 2 moderator**			
Task difficulty	−0.01	0.01	1.31
Thought control	0.003	0.01	0.41
Task difficulty x thought control	0.0002	0.0003	0.54
Deviance drop compared to unconditional model			493.88
Significance of likelihood ratio test			*p* < 0.001
**Model 1d—EBQ negative usefulness scale as level 2 moderator**			
Task difficulty	0.0008	0.01	0.11
Thought control	0.02	0.01	2.46^*^
Task difficulty x thought control	−0.0001	0.0004	0.22
Deviance drop compared to unconditional model			494.5
Significance of likelihood ratio test			*p* < 0.001
**Model 1e - PTQ score as level 2 moderator**			
Task difficulty	0.0001	0.003	0.31
Thought control	−0.01	0.004	1.36
Task difficulty x thought control	0.0001	0.0001	0.44
Deviance drop compared to unconditional model			497.74
Significance of likelihood ratio test			*p* < 0.001

TUT intensity is the outcome in all of the models.

DDFS—Daydreaming Frequency Scale, PTQ—Perseverative Thinking Questionnaire, EBQ—Emotional Beliefs Questionnaire.

* *p* < 0.05.

**Figure 4:**
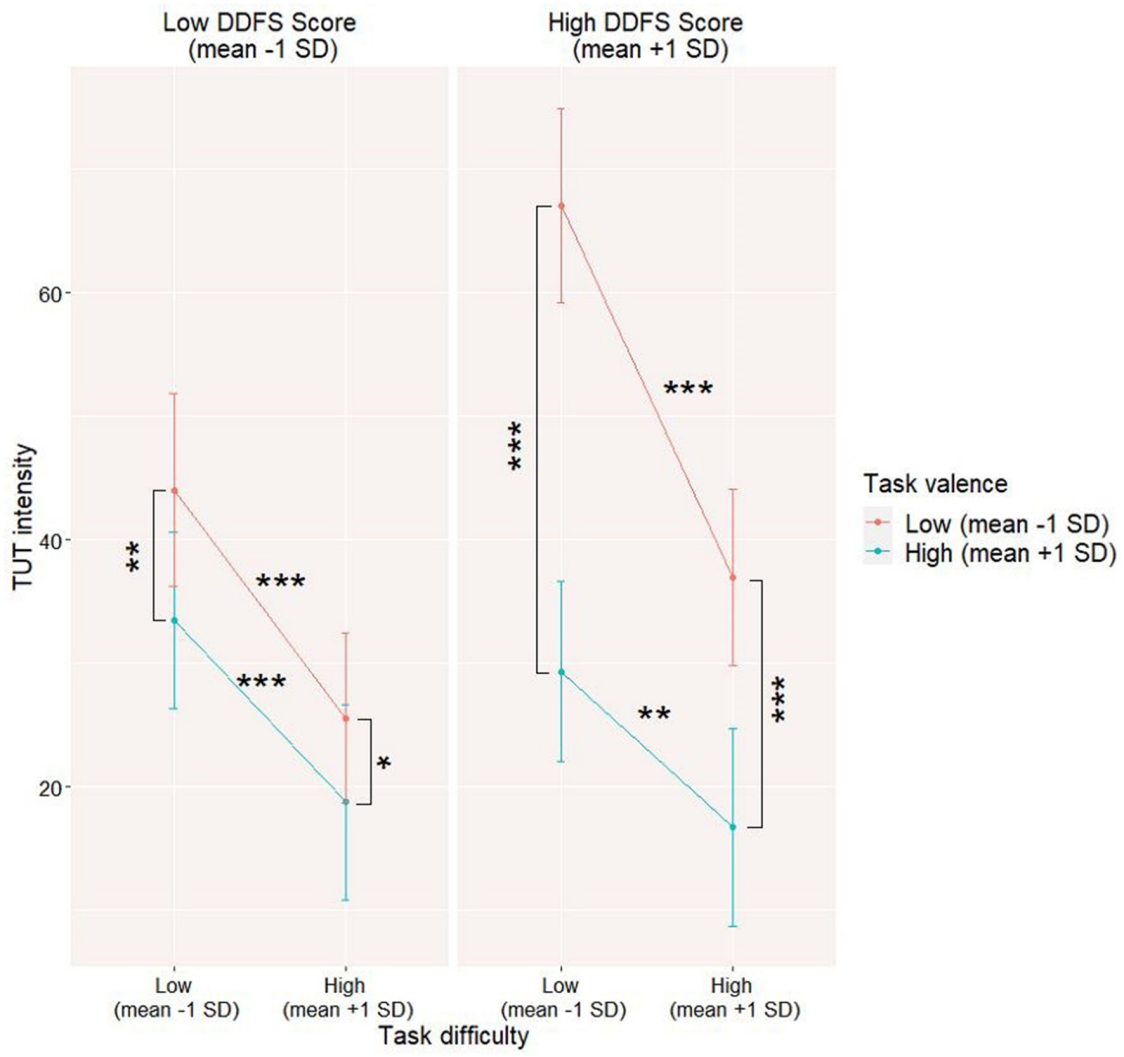
Daydreaming Frequency Scale (DDFS) moderating level 1 interaction between task valence and task difficulty on task-unrelated thoughts (TUT) level. The data for the moderation model were visualized by computing slopes for high (mean +1SD) and low (mean-1SD) levels of each predictor and moderator. *p < 0.05; **p < 0.01; ***p < 0.001.

Then, we constructed the models testing the moderation of the task difficulty x task valence interaction by trait-level characteristics. DDFS score significantly moderates the task difficulty x task valence interaction (refer to Model 2a in Table 4). The results suggest that the interaction between task difficulty and task valence can be stronger for people with higher levels of a trait tendency to daydream (refer to Figure 4).

**Table 4:** Testing level 2 variables as moderators of level 1 interactions in Model 2. TUT intensity is the outcome in all of the models.

**Models based on model 2**			
	***Coeff***	***SE***	***t-*** **ratio**
**Model 2a—DDFS score as level 2 moderator**			
Task difficulty	−0.005	0.004	1.30
Task valence	−0.02	0.0004	5.78^***^
Task difficulty x task valence	0.0003	0.0001	2.06^*^
Deviance drop compared to unconditional model			185.73
Significance of likelihood ratio test			*p* < 0.001
**Model 2b—EBQ score as level 2 moderator**			
Task difficulty	−0.01	0.003	2.36^*^
Task valence	−0.04	0.004	1.01
Task difficulty x task valence	0.0001	0.0001	1.03
Deviance drop compared to unconditional model			152.91
Significance of likelihood ratio test			*p* < 0.001
**Model 2c—EBQ negative controllability score as level 2 moderator**			
Task difficulty	−0.03	0.01	3.37^**^
Task valence	−0.02	0.01	1.87
Task difficulty x task valence	0.0007	0.0003	2.05^*^
Deviance drop compared to unconditional model			165.97
Significance of likelihood ratio test			*p* < 0.001
**Model 2d—EBQ negative usefulness score as level 2 moderator**			
Task difficulty	0.006	0.01	0.76
Task valence	−0.01	0.01	1.05
Task difficulty x task valence	−0.0001	0.0004	0.25
Deviance drop compared to unconditional model			148.19
Significance of likelihood ratio test			*p* < 0.001
**Model 2e—PTQ score as level 2 moderator**			
Task difficulty	−0.006	0.004	1.66
Task valence	−0.007	0.004	1.59
Task difficulty x task valence	−0.0001	0.0001	0.69
Deviance drop compared to unconditional model			160.08
Significance of likelihood ratio test			*p* < 0.001

DDFS—Daydreaming Frequency Scale, PTQ—Perseverative Thinking Questionnaire, EBQ—Emotional Beliefs Questionnaire.

* *p* < 0.05;

** *p* < 0.01;

*** *p* < 0.001.

**Figure 5:**
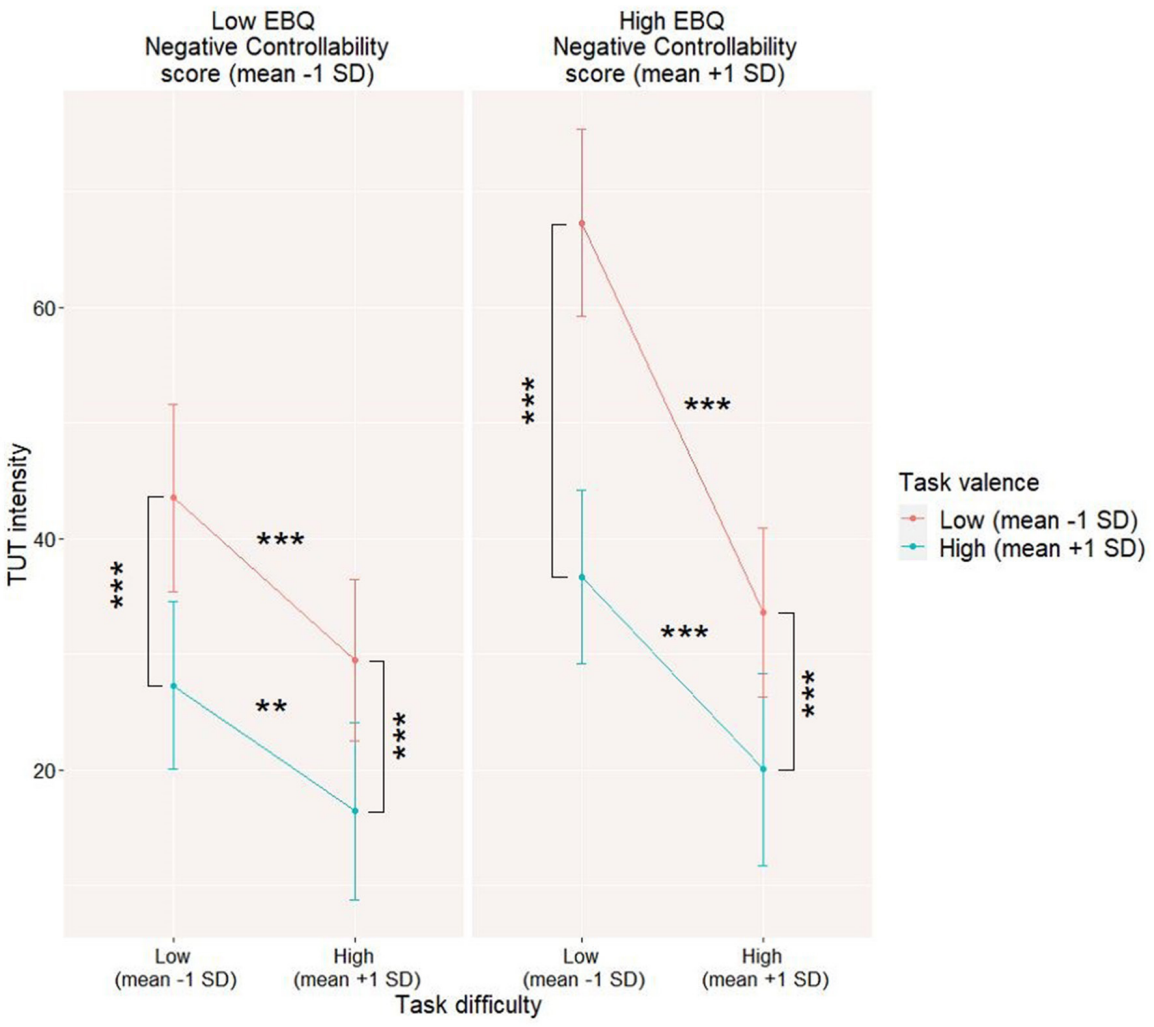
Emotion Beliefs Questionnaire (EBQ)—negative emotions controllability moderating level 1 interaction between thought control and task difficulty on task-unrelated thoughts (TUT) level. The data for the moderation model were visualized by computing slopes for high (mean +1SD) and low (mean-1SD) levels of each predictor and moderator. **p < 0.01; ***p < 0.001.

The total score of EBQ does not moderate the analyzed interaction (refer to Model 2b in Table 4); however, the negative controllability scale score of EBQ turned out to be a significant moderator of it (refer to Model 2c). The results suggest that stronger metacognitive beliefs about the uncontrollability of negative emotions can be associated with a stronger interaction between task difficulty and task valence (refer to Figure 5).

## Discussion

The main aim of the present study was to test whether avoidance mechanisms can be an alternative explanation to self-control failure for the high level of maladaptive task-unrelated thoughts (i.e., task-unrelated thought occurring when the ongoing task is difficult and one should fully focus on it). To the best of our knowledge, this is the first attempt of testing this hypothesis in a quantitative, experience sampling study. Determining the precise mechanisms of maladaptive TUT occurrence and maintenance seems to be crucial from the clinical perspective, as the literature suggests clearly that in spite of its numerous adaptive functions [e.g., (5–8)], TUT might also have maladaptive consequences, from impairing cognitive performance to emotion deregulation and increased risk of psychological disorders (7, 9, 11, 58). One of the hypotheses linking the contextual factors to TUT occurrence is the context regulation hypothesis (2), suggesting that a high level of TUT while performing cognitively demanding tasks might be due to self-control failure and the impossibility to inhibit off-task thinking. However, some empirical studies did not find support for this hypothesis suggesting that, in spite of good executive resources, participants can experience a high level of TUT during difficult tasks (21). An alternative hypothesis to explain the high level of TUT while performing a cognitively demanding task is TUT being an avoidance of a current difficult experience, but this hypothesis, in spite of a good theoretical founding [e.g., (59)], was only tested in qualitative studies (12, 15). The results of our study shed some new light on both potential mechanisms.

First, from the perspective of the context regulation hypothesis, we showed that both task difficulty and one's control over the thoughts significantly decrease the TUT level in participants' daily life. Moreover, the interaction between task difficulty and control over one's thoughts was a significant predictor of TUT level. It seems that in general, participants with lower subjective control of thoughts present a higher level of TUT. In spite of the fact that when they are performing a difficult task, the level of TUT decreases more in those participants compared to participants with a higher level of thought control, the level of TUT in participants with low control remains, however, significantly higher in general. Thus, it seems that participants with better subjective control over their thoughts can better manage TUT during a cognitively demanding task. These results complete the results of previous laboratory studies [e.g., (13, 14)] by testing the relation between task difficulty and control over thoughts in ecological settings. Although the impact of executive functions measured in the laboratory on experience sampling measures of TUT was previously shown by Marcusson-Clavertz et al. (19), to the best of our knowledge, only one study measured both task difficulty and thought control in participants' daily life and found an interaction between those factors in their impact on TUT level (51). However, those results are still limited by the fact that one's control over thoughts was only measured through self-reported assessment.

A similar pattern of results was found for the model testing the avoidant alternative mechanisms of TUT. Once again, it seems that participants report a higher level of TUT when the task is easy compared to a difficult one. Moreover, the TUT level is generally higher when participants are performing unpleasant tasks. Although the TUT level drops for both pleasant and unpleasant difficult tasks, the TUT level for difficult unpleasant tasks remains significantly higher compared to the pleasant ones. Thus, task valence can also play a key role in the TUT occurrence, suggesting that TUT can be used as an escape from unpleasant, difficult tasks, corroborating the results of previous studies showing that TUT might be triggered by negative affect [e.g., (30)].

It is also interesting to note that not only the trait tendency to use daydreaming might moderate the link between task valence and task difficulty on their impact on momentary TUT level but also the role of a trait moderator might be affected by one's beliefs on emotion and particularly on the possibility of controlling one's negative emotions. Surprisingly, those beliefs affect particularly TUT levels while performing unpleasant but easy tasks. The involvement of beliefs on emotion in the occurrence and maintenance of off-task thinking serving as an avoidance strategy is particularly important, as previous studies showed that beliefs on uncontrollability and lack of usefulness of emotions might lead to greater use of avoidance strategies (60).

Although the present study is the first to empirically test the avoidance hypothesis through the daily sampling method and shows that task valence and participants' beliefs on emotion play a significant role in TUT maintenance and its adaptive feature, some important questions remain unanswered and need to be addressed in the further studies. First, in the experience sampling methods, it is difficult to test simultaneously and detangle the effect of task valence and TUT on participants' affect, as affect and task valence are closely correlated—in the present study the correlation coefficients between affect variables (“happy,” “interested,” “sad,” “angry,” and “anxious”) and task valence had absolute values ranging from 0.30 to 0.61 (*p* < 0.001 for all). One of the possible solutions is to test the effect of TUT as avoidant behavior on lagged affect; however, as suggested by the theoretical models of anxiety disorders, it is possible that TUT as an avoidant emotion regulation strategy might have a paradoxical effect of increasing negative affect in the next measure point (59). Thus, it seems necessary to design an experimental study and control for the task valence.

Second, in the experience sampling part of the present study, we purposefully asked the questions about activity/task characteristics and not the particular task participants were exercising at the given moment. According to our hypothesis and previous studies [e.g., (51)], it is not the task type itself but the task characteristics that seem to be crucial in TUT occurrence. This differentiation between activity type and characteristics might be important particularly in the context of ecological assessment—the same/similar task but performed in the other context or by another individual might have different subjective valence and difficulty. However, this methodological decision found on theoretical reasons might be an additional source of variance in the present study as we were not able to control the type of activity participants were performing at the time of sampling. This issue also stresses that in order to fully test the avoidant alternative hypothesis of TUT, it is necessary to merge both experience sampling and controlled laboratory studies.

Third, future studies may go beyond the self-reported assessment of the control over one's thoughts to merge experience sampling methods with an objective measure of cognitive control through tasks measuring executive functions. Finally, further studies may test whether and how both hypotheses of TUT occurrence—the context regulation and avoidant alternative, might be related to each other.

In addition, it is necessary to underline that the results of the present study should be treated as preliminary. First, the sample size is relatively low (49 participants in the final analysis). Moreover, the study was run fully online and without paying the participants resulting in a considerable dropout between the trait and experience sampling part of the study (10 participants). In total, 13 participants needed to be excluded due to a low compliance rate in experience sampling [lower than 30%; (40–43)]. Second, although we tested some potential trait-level moderators of the presented models (the ones classically used in TUT studies—like propensity to daydream or use repetitive negative thinking and the one linked directly to the avoidant alternative hypothesis—beliefs on emotions), it seems important to note that also other personality level variables are suggested by the literature to affect the level of TUT [e.g., neuroticism, (13)] and should be included in further studies.

In spite of those shortcomings and the general difficulty to assess avoidance outside the lab, we believe that studying TUT function is crucial from the clinical perspective. First, studies testing avoidance in anxiety disorders bring some evidence that this mechanism might be a key element in the maintenance of psychopathology (35). Second, there is an ongoing debate about whether we should consider maladaptive daydreaming as a psychological disorder or a potential transdiagnostic risk factor (11). Thus, it seems crucial to understand not only what mechanism is responsible for the occurrence of maladaptive TUT but also what mechanism(s) should be taken into account and addressed during the therapeutic process. According to the results of our study, while addressing maladaptive TUT both in research and clinical settings, one should not only focus on the contextual factors like task difficulty and patients' executive functioning but also mechanisms linked to emotion regulation like experiential avoidance and metacognitive beliefs on emotion. Although more research is needed to support the mechanism of avoidance in TUT and to explore the role of beliefs on emotions, we believe this study is one of the first important indicators bringing preliminary empirical evidence that these factors might matter in the maladaptive task-unrelated thoughts.

## Data availability statement

The original contributions presented in the study are included in the article/Supplementary material, further inquiries can be directed to the corresponding author.

## Ethics statement

The studies involving human participants were reviewed and approved by Research Ethics Commission, SWPS University of Social Sciences and Humanities, Katowice, Poland. The participants provided their written informed consent to participate in this study.

## Author contributions

MK: conceptualization, methodology, writing—original draft, and supervision of the project. MS: formal analysis, data curation, visualization, and writing—original draft. IK: writing—review and editing. All authors contributed to the article and approved the submitted version.

## Footnotes

^1^A total of 10 participants who did not fully complete the trait-level evaluation on day 1 were contacted after the end of the experience sampling part of the study and completed the questionnaires at that time.

## References

[1] Marcusson-Clavertz D Cardeña E Terhune DB. Daydreaming style moderates the relation between working memory and mind wandering: Integrating two hypotheses. J Exp Psychol Learn Mem Cogn. (2016) 42:451–64. 10.1037/xlm0000180 26371497

[2] Kane MJ McVay JC. What mind wandering reveals about executive-control abilities and failures. Curr Dir Psychol Sci. (2012) 21:348–54. 10.1177/0963721412454875

[3] Klinger E Cox WM. Dimensions of thought flow in everyday life. Imagin Cogn Pers. (1987) 7:105–28. 10.2190/7K24-G343-MTQW-115V19999275

[4] Seli P Beaty RE Cheyne JA Smilek D Oakman J Schacter DL. How pervasive is mind wandering really? Conscious Cogn. (2018) 66:74–8. 10.1016/j.concog.2018.10.002 30408603

[5] Klinger E. Goal Commitments and the content of thoughts and dreams: basic principles. Front Psychol. (2013) 4:415. 10.3389/fpsyg.2013.00415 23874312 PMC3708449

[6] Klinger E Koster EHW Marchetti I. Spontaneous thought and goal pursuit: from functions such as planning to dysfunctions such as rumination. In:Christoff K. Fox KCR, editors. The Oxford Handbook of Spontaneous Thought: Mind-Wandering, Creativity, and Dreaming. Oxford: Oxford Library of Psychology (2018). p. 215–32

[7] Mooneyham BW Schooler JW. The costs and benefits of mind-wandering: a review. Can J Exp Psychol Rev. (2013) 67:11–8. 10.1037/a0031569 23458547

[8] Stawarczyk D Majerus S Maj M Van der Linden M D'Argembeau A. Mind-wandering: phenomenology and function as assessed with a novel experience sampling method. Acta Psychol. (2011) 136:370–81. 10.1016/j.actpsy.2011.01.002 21349473

[9] Mar RA Mason MF Litvack A. How daydreaming relates to life satisfaction, loneliness, and social support: the importance of gender and daydream content. Conscious Cogn. (2012) 21:401–7. 10.1016/j.concog.2011.08.001 22033437

[10] Marchetti I Van de Putte E Koster EHW. Self-generated thoughts and depression: From daydreaming to depressive symptoms. Front Hum Neurosci. (2014) 8:131. 10.3389/fnhum.2014.00131 24672458 PMC3957030

[11] Schimmenti A Somer E Regis M. Maladaptive daydreaming: towards a nosological definition. Annales Médico-Psychologiques, Revue Psychiatrique. (2019) 177:865–74. 10.1016/j.amp.2019.08.014

[12] Somer E. Maladaptive daydreaming: a qualitative inquiry. J Contemp Psychother. (2002) 32:197–212. 10.1023/A:1020597026919

[13] Robison MK Gath KI Unsworth N. The neurotic wandering mind: an individual differences investigation of neuroticism, mind-wandering, and executive control. Q J Exp Psychol. (2017) 70:649–63. 10.1080/17470218.2016.1145706 26821933

[14] Rummel J Boywitt CD. Controlling the stream of thought: working memory capacity predicts adjustment of mind-wandering to situational demands. Psychon Bull Rev. (2014) 21:1309–15. 10.3758/s13423-013-0580-3 24448763

[15] Pietkiewicz IJ Necki S Bańbura A Tomalski R. Maladaptive daydreaming as a new form of behavioral addiction. J Behav Addict. (2018) 7:838–43. 10.1556/2006.7.2018.95 30238787 PMC6426361

[16] Kane MJ Gross GM Chun CA Smeekens BA Meier ME Silvia PJ. For whom the mind wanders, and when, varies across laboratory and dailylife settings. Psychol Sci. (2017) 28:1271–89. 10.1177/0956797617706086 28719760 PMC5591044

[17] Levinson DB Smallwood J Davidson RJ. The persistence of thought: evidence for a role of working memory in the maintenance of task-unrelated thinking. Psychol Sci. (2012) 23:375–80. 10.1177/0956797611431465 22421205 PMC3328662

[18] McVay JC Kane MJ. Conducting the train of thought: working memory capacity, goal neglect, and mind wandering in an executive-control task. J Exp Psychol Learn Mem Cogn. (2009) 35:196–204. 10.1037/a0014104 19210090 PMC2750806

[19] Marcusson-Clavertz D Persson SD Cardeña E. The contribution of latent factors of executive functioning to mind wandering: an experience sampling study. Cogn Res Princ Implic. (2022) 7:34. 10.1186/s41235-022-00383-9 35467232 PMC9038971

[20] Barrington M Miller L Roodenrys S. Integrating the mind wandering-task difficulty relationship: the determinants of working memory, intentionality, motivation, and subjective difficulty. SSRN Elect J. (2022). 10.2139/ssrn.4062450

[21] Randall JG Beier ME Villado AJ. Multiple routes to mind wandering: predicting mind wandering with resource theories. Conscious Cogn. (2019) 67:26–43. 10.1016/j.concog.2018.11.006 30502635

[22] Christoff K Mills C Andrews-Hanna JR Irving ZC Thompson E Fox KCR. Mind-wandering as a scientific concept: cutting through the definitional Haze. Trends Cogn Sci. (2018) 22:957–9. 10.1016/j.tics.2018.07.004 30220476

[23] Ottaviani C Shapiro D Couyoumdjian A. Flexibility as the key for somatic health: From mind wandering to perseverative cognition. Biol Psychol. (2013) 94:38–43. 10.1016/j.biopsycho.2013.05.003 23680439

[24] DuPre E Spreng NR. (2018). Rumination Is a Sticky Form of Spontaneous Thought. In:Christoff K Fox KCR, editors. The Oxford Handbook of Spontaneous Thought: Mind-Wandering, Creativity, and Dreaming. Oxford: Oxford Library of Psychology. p. 509–20.

[25] Klinger E Cox WM. Motivation and the Goal Theory of Current Concerns. In: Klinger E, Cox WM, editors. Handbook of Motivational Counseling: Goal-Based Approaches to Assessment and Intervention with Addiction and Other Problems. Hoboken, NJ: Wiley Blackwell (2011). p. 3–47.

[26] van Rijn E Reid AM Edwards CL Malinowski JE Ruby PM Eichenlaub JB. Daydreams incorporate recent waking life concerns but do not show delayed ('dream-lag') incorporations. Conscious Cogn. (2018) 58:51–9. 10.1016/j.concog.2017.10.011 29128282

[27] Martin LL Tesser A. Some ruminative thoughts. In Wyer RS, editor. Ruminative Thoughts: Advances in Social Cognition. Lawrence Erlbaum Associates, Inc (1996). p. 1–47.

[28] Borkovec TD Alcaine OM Behar E. Avoidance theory of worry and generalized anxiety disorder. In:Heimberg RG Turk CL Mennin DS, editors. Generalized Anxiety Disorder: Advances in Research and Practice. The Guilford Press (2004). p. 77–108.

[29] Giorgio JM Sanflippo J Kleiman E Reilly D Bender RE Wagner CA. An experiential avoidance conceptualization of depressive rumination: three tests of the model. Behav Res Ther. (2010) 48:1021–31. 10.1016/j.brat.2010.07.004 20691426 PMC3045819

[30] Poerio GL Totterdell P Miles E. Mind-wandering and negative mood: does one thing really lead to another? Conscious Cogn. (2013) 22:1412–21. 10.1016/j.concog.2013.09.012 24149091

[31] Kane MJ Brown LH McVay JC Silvia PJ Myin-Germeys I Kwapil TR. For whom the mind wanders, and when: an experience-sampling study of working memory and executive control in daily life. Psychol Sci. (2007) 18:614–21. 10.1111/j.1467-9280.2007.01948.x 17614870

[32] Crosswell AD Coccia M Epel ES. Mind wandering and stress: when you don't like the present moment. Emotion. (2020) 20:403–12. 10.1037/emo0000548 30714780 PMC6679812

[33] Ford BQ Gross JJ. Why beliefs about emotion matter: an emotion-regulation perspective. Curr Dir Psychol Sci. (2019) 28:74–81. 10.1177/0963721418806697

[34] Ottenbreit ND Dobson KS. Avoidance and depression: the construction of the Cognitive–Behavioral Avoidance Scale. Behav Res Ther. (2004) 42:293–313. 10.1016/S0005-7967(03)00140-2 14975771

[35] Loijen A Vrijsen JN Egger JIM Becker ES Rinck M. Biased approach-avoidance tendencies in psychopathology: a systematic review of their assessment and modification. Clin Psychol Rev. (2020) 77:101825. 10.1016/j.cpr.2020.101825 32143108

[36] Trull TJ Ebner-Priemer UW. Using experience sampling methods/ecological momentary assessment (ESM/EMA) in clinical assessment and clinical research: introduction to the special section. Psychol Assess. (2009) 21:457–62. 10.1037/a0017653 19947780 PMC4255457

[37] Ellison WD Trahan AC Pinzon JC Gillespie ME Simmons LM King KY. For whom, and for what, is experience sampling more accurate than retrospective report? Pers Individ Dif. (2020) 163:110071. 10.1016/j.paid.2020.110071

[38] movisens GmbH,. MovisensXS. (2020). Available online at: https://www.movisens.com/en/products/movisensxs/ (accessed January 22, 2023).

[39] Carter LA. Rigorous Methods for the Analysis, Reporting and Evaluation of ESM Style Data. Doctoral dissertation. Manchester: The University of Manchester (2016).

[40] Rintala A Apers S Eisele G Verboeven D. Briefing and debriefing in an experience sampling study. In Myin-Germeys I, Kuppens P, editors. The Open Handbook of Experience Sampling Methodology: A Step-by-Step Guide to Designing, Conducting, and Analyzing ESM Studies. Leuven: Center for Research on Experience Sampling and Ambulatory Methods Leuven (2022). p. 119–34.

[41] Ramsey AT Wetherell JL Depp C Dixon D Lenze E. Feasibility and acceptability of smartphone assessment in older adults with cognitive and emotional difficulties. J Technol Hum Serv. (2016) 34:209–23. 10.1080/15228835.2016.1170649 27683018 PMC5036573

[42] Wichers M Lothmann C Simons CJ Nicolson NA Peeters F. The dynamic interplay between negative and positive emotions in daily life predicts response to treatment in depression: a momentary assessment study. Br J Clin Psychol. (2012) 51:206–22. 10.1111/j.2044-8260.2011.02021.x 22574805

[43] Henquet C Van Os J Kuepper R Delespaul P Smits M Campo J. Psychosis reactivity to cannabis use in daily life: an experience sampling study. Br J Psychiatry. (2010) 196:447–53. 10.1192/bjp.bp.109.072249 20513854

[44] Rintala A Wampers M Myin-Germeys I Viechtbauer W. Response compliance and predictors thereof in studies using the experience sampling method. Psychol Assess. (2019) 31:226–35. 10.1037/pas0000662 30394762

[45] Giambra LM. The influence of aging on spontaneous shifts of attention from external stimuli to the contents of consciousness. Exp Gerontol. (1993) 28:485–92. 10.1016/0531-5565(93)90073-M 8224044

[46] Singer JL Antrobus JS. Manual for the Imaginal Process Inventory. Princeton: Educational Testing Service (1970).

[47] Singer JL Antrobus JS. Daydreaming, imaginal process and personality: a normative study. In:Sheehan PW, editor. The Function and Nature of Imagery. New York, NY: Academic Press (1972). p.175–202.

[48] Skorupski M Krejtz I Kornacka M. The Assessment of Daydreaming Frequency and Control With the Polish Version of Daydreaming Frequency Scale – A Validation Study Using Ecological Momentary Assessment. (2023). 10.3389/fpsyt.2025.1694756 PMC12914265 41716869

[49] Ehring T Zetsche U Weidacker K Wahl K Schönfeld S Ehlers A. The perseverative thinking questionnaire (PTQ): validation of a content-independent measure of repetitive negative thinking. J Behav Ther Exp Psychiatry. (2011) 42:225–32. 10.1016/j.jbtep.2010.12.003 21315886 PMC3042595

[50] Becerra R Preece DA Gross JJ. Assessing beliefs about emotions: development and validation of the emotion beliefs questionnaire. PLoS ONE. (2020) 15:e0231395. 10.1371/journal.pone.0231395 32287328 PMC7156043

[51] Kornacka M Atzeni T Krejtz I Bortolon C Baeyens C. Task unrelated thoughts (TUT) affecting mood in ecological settings: from adaptive mind-wandering to maladaptive rumination. In: Proceedings of the Annual Meeting of the Cognitive Science Society. (2022). p. 44. Available online at: https://escholarship.org/uc/item/8372r3nr

[52] Granholm E Holden JL Mikhael T Link PC Swendsen J Depp C. What do people with schizophrenia do all day? Ecological momentary assessment of real-world functioning in Schizophrenia. Schizophr Bull. (2020) 46:242–51. 10.1093/schbul/sbz070 31504955 PMC7442321

[53] Pe ML Vandekerckhove J Kuppens P. A diffusion model account of the relationship between the emotional flanker task and rumination and depression. Emotion. (2013) 13:739–47. 10.1037/a0031628 23527499

[54] R Core Team. R: A Language Environment for Statistical Computing. Vienna, Austria (2022). Available online at: https://www.R-project.org/ (accessed January 22, 2023).

[55] Bates D Mächler M Bolker B Walker S. Fitting linear mixed-effects models using lme4. J Stat Softw. (2015) 67:1–48. 10.18637/jss.v067.i01

[56] Johnston R Jones K Manley D. Confounding and collinearity in regression analysis: a cautionary tale and an alternative procedure, illustrated by studies of British voting behaviour. Qual Quan. (2018) 52:1957–76. 10.1007/s11135-017-0584-6 29937587 PMC5993839

[57] Gareth J Daniela W Trevor H Robert T. An introduction to statistical learning: with applications in R. Spinger. (2013).12792624

[58] Marchetti I Koster EHW Klinger E Alloy LB. Spontaneous thought and vulnerability to mood disorders: the dark side of the wandering mind. Clin Psychol Sci. (2016) 4:835–57. 10.1177/2167702615622383 28785510 PMC5544025

[59] Borkovec TD Hazlett-Stevens H Diaz ML. The role of positive beliefs about worry in generalized anxiety disorder and its treatment. Clin Psychol Psychother. (1999) 6:126–38. 10.1002/(SICI)1099-0879(199905)6:2<126::AID-CPP193>3.0.CO;2-M

[60] De Castella K Platow MJ Tamir M Gross JJ. Beliefs about emotion: implications for avoidance-based emotion regulation and psychological health. Cogn Emot. (2018) 32:773–95. 10.1080/02699931.2017.1353485 28737108

