# Supplementary material for: Maladaptive task-unrelated thoughts: Self-control failure or avoidant behavior? Preliminary evidence from an experience sampling study
Source: Front Psychiatry. 2023 Mar 14;14:1037443. doi: 10.3389/fpsyt.2023.1037443 (PMC10043255; doi:10.3389/fpsyt.2023.1037443)
Supplement: Supplementary file 1 [file Table_1.DOCX]

# **Supplementary material**

We present above the models computed for the full sample of participants taking part in EMA, independently of the compliance rate (only participants with 1 EMA response were excluded). The full sample was composed of 56 people (*Mean age* = 30.68, *SD* = 5.79, 42.1% female) with the mean compliance rate of 59,30%.

# **Supplementary material table 1.**

# *Descriptive Statistics of Level 1 and 2 variables.*

| Level 1 variables (N=56) | | |
| --- | --- | --- |
| Variable | Mean | SD |
| TUT intensity | 33.32 | 32.89 |
| Thought control | 69.01 | 28.00 |
| TUT valence | 71.25 | 26.39 |
| Task difficulty | 26.37 | 29.18 |
| Task interesting | 58.85 | 32.27 |
| Task pleasant | 67.64 | 26.72 |
| Level 2 variables (N=56) | | |
| DDFS | 38.44 | 9.20 |
| PTQ | 44.31 | 9.81 |
| EBQ total score | 31.07 | 11.95 |
| EBQ negative controllability | 9.11 | 3.97 |
| EBQ positive controllability | 9.71 | 4.95 |
| EBQ negative usefulness | 7.62 | 4.63 |
| EBQ positive usefulness | 4.85 | 2.34 |

Note. DDFS - Daydreaming Frequency Scale, PTQ - Perseverative Thinking Questionnaire, EBQ- Emotional Beliefs Questionnaire.

**Supplementary material table 2**

*Testing level 1 predictors and their interactions link to momentary TUT intensity.*

|  |  | *Coeff* | *SE* | *t-value* |
| --- | --- | --- | --- | --- |
| Model 1 – Task difficulty and thought control interaction | | |  |  |
|  | Task difficulty | -0.17 | 0.03 | 5.52*** |
|  | Thought control | -0.78 | 0.03 | 23.35*** |
|  | Task difficulty x Thought control | 0.003 | 0.001 | 2.12* |
| Deviance drop compared to unconditional model | | | | 493.13 |
| Significance of likelihood ratio test | | | | *p* < .001 |
| Model 2 – Task difficulty and task valence interaction | | | |  |
|  | Task difficulty | -0.37 | 0.04 | 10.21*** |
|  | Task valence | -0.40 | 0.04 | 10.02*** |
|  | Task difficulty x Task valence | 0.004 | 0.001 | 3.06** |
| Deviance drop compared to unconditional model | | | | 159.23 |
| Significance of likelihood ratio test | | | | *p* < .001 |

**p*<.05; ***p*<.01; ****p*<.001

**Supplementary material table 3.**

*Testing level 2 variables as moderators of level 1 interactions in Model 1. TUT intensity is the outcome in all of the models.*

| Models based on Model 1 | | | | |
| --- | --- | --- | --- | --- |
|  |  | *Coeff* | *SE* | *t-ratio* |
| Model 1a – DDFS score as level 2 moderator | | | |  |
|  | Task difficulty | 0.004 | 0.003 | 1.18 |
|  | Thought control | -0.005 | 0.004 | 1.55 |
|  | Task difficulty x Thought control | -0.0003 | 0.0001 | 1.88 |
| Deviance drop compared to unconditional model | | | | 501.1 |
| Significance of likelihood ratio test | | | | *p* < .001 |
| Model 1b – EBQ score as level 2 moderator | | | |  |
|  | Task difficulty | -0.003 | 0.003 | 0.93 |
|  | Thought control | 0.005 | 0.003 | 1.59 |
|  | Task difficulty x Thought control | 0.0001 | 0.0001 | 0.97 |
| Deviance drop compared to unconditional model | | | | 488.5 |
| Significance of likelihood ratio test | | | | *p* < .001 |
| Model 1c – EBQ Negative Controllability score as level 2 moderator | | | |  |
|  | Task difficulty | -0.01 | 0.008 | 1.28 |
|  | Thought control | 0.006 | 0.008 | 0.73 |
|  | (Task difficulty x Thought control) | 0.0001 | 0.0003 | 0.42 |
| Deviance drop compared to unconditional model | | | | 488.72 |
| Significance of likelihood ratio test | | | | *p* < .001 |
| Model 1d – EBQ Negative Usefulness scale as level 2 moderator | | | |  |
|  | Task difficulty | 0.0004 | 0.007 | 0.06 |
|  | Thought control | 0.02 | 0.009 | 2.34* |
|  | (Task difficulty x Thought control) | 0.000001 | 0.0003 | 0.34 |
| Deviance drop compared to unconditional model | | | | 485.38 |
| Significance of likelihood ratio test | | | | *p* < .001 |
| Model 1e - PTQ score as level 2 moderator | | | |  |
|  | Task difficulty | -0.0001 | 0.003 | 0.003 |
|  | Thought control | -0.003 | 0.004 | 0.74 |
|  | (Task difficulty x Thought control) | -0.0001 | 0.0001 | 0.60 |
| Deviance drop compared to unconditional model | | | | 503.56 |
| Significance of likelihood ratio test | | | | *p* < .001 |

*Note.* DDFS - Daydreaming Frequency Scale, PTQ - Perseverative Thinking Questionnaire, EBQ- Emotional Beliefs Questionnaire.

**p*<.05; ***p*<.01; ****p*<.001

**Supplementary material Table 4.**

*Testing level 2 variables as moderators of level 1 interactions in Model 2. TUT intensity is the outcome in all of the models.*

| Models based on Model 2 | | | | |
| --- | --- | --- | --- | --- |
|  |  | *Coeff* | *SE* | *t-ratio* |
| Model 2a – DDFS score as level 2 moderator | | | |  |
|  | Task difficulty | -0.005 | 0.004 | 1.36 |
|  | Task valence | -0.02 | 0.004 | 5.79*** |
|  | (Task difficulty x Task valence) | 0.0003 | 0.0001 | 1.98* |
| Deviance drop compared to unconditional model | | | | 199.11 |
| Significance of likelihood ratio test | | | | *p*<.001 |
| Model 2b – EBQ score as level 2 moderator | | | |  |
|  | Task difficulty | -0.007 | 0.003 | 2.28* |
|  | Task valence | -0.002 | 0.004 | 0.65 |
|  | (Task difficulty x Task valence) | 0.0001 | 0.0001 | 0.86 |
| Deviance drop compared to unconditional model | | | | 162.57 |
| Significance of likelihood ratio test | | | | *p*<.001 |
| Model 2c – EBQ Negative Controllability score as level 2 moderator | | | |  |
|  | Task difficulty | -0.03 | 0.01 | 3.22* |
|  | Task valence | -0.02 | 0.01 | 1.53 |
|  | (Task difficulty x Task valence) | 0.0006 | 0.0003 | 1.81 |
| Deviance drop compared to unconditional model | | | | 167.44 |
| Significance of likelihood ratio test | | | | *p*<.001 |
| Model 2d – EBQ Negative Usefulness score as level 2 moderator | | | |  |
|  | Task difficulty | 0.005 | 0.01 | 0.62 |
|  | Task valence | -0.07 | 0.01 | 0.79 |
|  | (Task difficulty x Task valence) | -0.0001 | 0.0003 | 0.27 |
| Deviance drop compared to unconditional model | | | | 156.72 |
| Significance of likelihood ratio test | | | | *p*<.001 |
| Model 2e – PTQ score as level 2 moderator | | | |  |
|  | Task difficulty | -0.006 | 0.004 | 1.69 |
|  | Task valence | -0.006 | 0.004 | 1.43 |
|  | (Task difficulty x Task valence) | -0.0001 | 0.0001 | 0.85 |
| Deviance drop compared to unconditional model | | | | 151.19 |
| Significance of likelihood ratio test | | | | *p*<.001 |

*Note.* DDF- Daydreaming Frequency Scale, PTQ- Perseverative Thinking Questionnaire, EBQ- Emotional Beliefs Questionnaire.

**p*<.05; ***p*<.01; ****p*<.001
